# Development of Digital Health Messages for Rural Populations in Tanzania: Multi- and Interdisciplinary Approach

**DOI:** 10.2196/25558

**Published:** 2021-09-22

**Authors:** Christine Holst, Ghislain Maurice Norbert Isabwe, Felix Sukums, Helena Ngowi, Flora Kajuna, Danica Radovanović, Wisam Mansour, Elibariki Mwakapeje, Peter Cardellichio, Bernard Ngowi, Josef Noll, Andrea Sylvia Winkler

**Affiliations:** 1 Centre for Global Health Department of Community Medicine and Global Health, Institute of Health and Society University of Oslo Oslo Norway; 2 Future Competence International Kigali Rwanda; 3 Department of Information and Communication Technology University of Agder Grimstad Norway; 4 Directorate of Information and Communication Technology Muhimbili University of Health and Allied Sciences Dar es Salaam United Republic of Tanzania; 5 Department of Veterinary Medicine and Public Health Sokoine University of Agriculture Morogoro United Republic of Tanzania; 6 Basic Internet Foundation Kjeller Norway; 7 Epidemiology and Diseases Control Section Ministry of Health, Community Development, Gender, Elderly and Children Dodoma United Republic of Tanzania; 8 Global Health Media Project Waitsfield, VT United States; 9 Muhimbili Medical Research Centre National Institute for Medical Research Dar es Salaam United Republic of Tanzania; 10 Mbeya College of Health and Allied Sciences University of Dar es Salaam Mbeya United Republic of Tanzania; 11 Department of Technology Systems University of Oslo Oslo Norway; 12 Center for Global Health, Department of Neurology Technical University of Munich Munich Germany

**Keywords:** digital health, eHealth, mHealth, Tanzania, health education, HIV/AIDS, tuberculosis, cysticercosis, tapeworm, anthrax, mobile phone

## Abstract

**Background:**

Health workers have traditionally delivered health promotion and education to rural communities in the Global South in paper leaflet formats or orally. With the rise of digital technologies, health promotion and education can be provided in innovative and more effective formats, which are believed to have a higher impact on disease prevention and treatment.

**Objective:**

The aim of this tutorial is to illustrate how a multi- and interdisciplinary approach can be applied in the design process of digital health messages for use in the Global South.

**Methods:**

The multi- and interdisciplinary team of the Non-discriminating access for Digital Inclusion (DigI) project digitalized and customized available government-approved paper-based health promotion messages into a screen-suitable format. The team worked closely together and used its diverse expertise to develop digital health messages with disease-specific content in Tanzania’s national language (Swahili) as well as English. The development process included the following phases: a local needs assessment; identification of government-approved health promotion materials in a nondigital format; identification of key health messages; creation of a practical and engaging story, easy to understand for the general public; drafting of a storyboard for an animated video with review, feedback, and revisions; forward and backward translation; audio recording of the story in both languages; finalization and presentation of the animations; development of relevant questions related to the health messages in each domain; and development of web and mobile apps to access the digital health messages.

**Results:**

Between 2017 and 2019, we developed key health messages, quizzes, and animated health videos to address HIV/AIDS, tuberculosis, Taenia solium cysticercosis and taeniasis, and anthrax, all of which are of public health importance in Tanzania. Feedback from local stakeholders and test users was included in various phases of the process. The 4 videos and other content are available in local information spots on a digital health platform (DigI platform), established by the DigI project, in both Tanzanian Swahili and English.

**Conclusions:**

Our methodological multi- and interdisciplinary approach ensures that the digital health messages for the public are clear, high quality, and align with the government’s objectives for health promotion. It also demonstrates the diversity of scientific disciplines required when collaborating on a digital health project. We recommend this approach to be applied to the development of other digital health messages for a wide range of diseases.

**International Registered Report Identifier (IRRID):**

RR2-10.2196/25128

## Introduction

### Background

The World Health Organization (WHO) emphasizes the use of digital technologies to enable people to access information, goods, and services to improve their lives [1]. Sustainable Development Goals number 3 (good health and well-being) [2] and number 9 (industries, innovation, and infrastructure) [3] are both linked to digital health. More specifically, this is reflected by strengthening the capacity for early warning, risk reduction, and management of national and global health risks (target 3.D) and significantly increasing access to information and communication technology (target 9.C). Digital health is connected to multiple disciplines and critically depends on the country and the health system context [4]. Multidisciplinary research is about coordinating efforts that bring several disciplines together to provide complementary contributions in the service of a common goal [5] and is essential in the progress of any high-quality cross-cutting research project today. Interdisciplinarity relates to analyzing, synthesizing, and harmonizing the efforts into a common coordinated and coherent entity [6], and has previously been defined as “a mode of research by teams or individuals that integrates information, data, techniques, tools, perspectives, concepts, and/or theories from two or more disciplines or bodies of specialized knowledge to advance fundamental understanding or to solve problems whose solutions are beyond the scope of a single discipline or area of research practice.” [7]

It is well known that health promotion and participation in health promotion activities will be revolutionized by digital innovations and that health literacy increases when people begin using digital tools to access information and make informed decisions related to their own health [8]. However, low levels of literacy and digital skills are barriers to accessing digital information, especially in rural areas of low-income countries [9]. Thus, it is important that the design of a digital solution is inclusive and ensures that users develop the skills needed to take advantage of such digital opportunities [10].

Designing digital health messages for a Global South audience can be challenging considering the abovementioned shortfalls in literacy, digital literacy, access to internet, and access to devices. Power structures, poverty, gender inequality, and health inequities at large, including poor infrastructure, complicate the situation for several groups, including those marginalized. However, the future of health education and health promotion participation in sub-Saharan Africa may very well be digital [11]. The importance of early partnering with local stakeholders and users in a co-design process is crucial for success [12]. In the next section, we will present our project exemplified by a case study from Tanzania, before illustrating how we developed content for a global digital health intervention using a multi- and interdisciplinary approach.

### The Non-Discriminating Access for Digital Inclusion Project: Digital Health Promotion and Education in Tanzania

In Tanzania, community health workers and health facilities, in addition to nongovernmental organizations, have traditionally provided health promotion and education orally or in printed format (leaflets, billboards, banners, posters, etc) to neighborhoods and communities [13]. The Tanzanian digital landscape is evolving rapidly, and together with significant economic growth [14], we have witnessed increasing penetration of mobile phones in both urban and rural areas and one of the most advanced mobile money markets in sub-Saharan Africa [15]. Tanzania, East Africa’s third largest economy with 54.2 million people, had 43.6 million mobile phone subscribers as of June 2018 (80% of the population), compared with 40.1 million a year earlier and in contrast to only 10.2 out of 40.7 million people (25% of the population) in 2007 [16]. However, only 13% of subscribers reported to own a smartphone in 2017 [17]. The Tanzania Digital Health Strategy 2019-2024 seeks to expand the use of digital health technologies to promote healthy behavior through access to health information, education, and communication [18]. The Tanzanian government intends to scale up and intensify the achievements of the previous eHealth Strategy 2013-2018, which implemented various electronic information systems (including the use of television, radio, social media, etc) to provide and promote health education. Although community-based health services focus on health promotion and disease prevention, mobile health and social media can be more widely used to provide quality health education, information, and communication, enabling communities to adopt healthier behaviors and to increase their health literacy. Impactful health messages are clearly needed in the global fight against infectious diseases such as HIV/AIDS, tuberculosis (TB), and zoonosis (diseases that affect animals and humans) such as Taenia solium cysticercosis and taeniasis (TSCT) and anthrax. The Tanzanian Health Sector Strategic Plan states that the disease control programs for HIV/AIDS and TB have been successful in the areas of early detection and treatment. However, further improvements are needed in the field of prevention [13]. As for TSCT, which is recognized as a neglected tropical disease by the WHO [19], prevention is crucial because the disease is highly endemic in Tanzania [20]. Anthrax is an emerging neglected zoonotic disease, endemic in the African region and present in the north of Tanzania (especially in the Maasai communities) where repeated outbreaks in livestock, wildlife, and humans have made clear the need to strengthen prevention strategies [21].

The Non-discriminating access for Digital Inclusion (DigI) project, in which the development of health messages described in this paper is part, represents a classical multi- and interdisciplinary project funded as an innovation project, with the aim of connecting rural villages to digital information. The DigI project’s multi- and interdisciplinary team, comprising 11 partners from 8 countries, includes scientists in the disciplines of humanities, social sciences, formal sciences, and applied sciences. These main disciplines include a variety of subdisciplines: medicine and health, epidemiology, public health, veterinary medicine, electronic engineering, visual and creative arts with graphic and interaction design, human-computer interaction, communication, education or educational technology, information science, internet science, and ethics. Elements from anthropology, human geography, health psychology, and sociology of health and illness have been applied in the various steps of planning and implementation of this project. Through our collaboration of Tanzanian, German, Norwegian, Rwandan, and American partners, our objective is to develop digital health messages by converting printed materials into digital formats such as animated videos (animations), quizzes, graphics, and texts. The majority of the DigI partners either belonged to the information technology (IT) and design task force, or the health task force. The Tanzanian and German project partners with backgrounds in health and medicine provided expertise on HIV/AIDS, TB, TSCT, and anthrax. The team was able to draw on their many years of experience working with each disease in the target areas. The selected diseases in the DigI project are endemic in the chosen geographical areas and thus are important diseases in the Tanzanian public health context. All these diseases are of high priority and require preventive strategies including information dissemination to communities and populations.

A digital health education platform, the DigI platform [22], herein referred to as *the platform* was planned and developed between 2017 and 2019 by the DigI team. It consists of a dashboard (Multimedia Appendix 1) where viewers can navigate between various pages with information about the diseases. The platform features texts, quizzes, graphics, and animated videos that are available for all. The platform with the health messages described in this paper was established as part of an information spot (InfoSpot) in the rural villages of Selela and Esilalei in the Arusha region (for anthrax) and Migoli and Izazi in the Iringa region (for HIV/AIDS, TB, and TSCT) in November 2019. It is possible to access the health messages on the platform, even if the user is not connected to the internet, because the platform is also locally stored on a village server.

In this tutorial, we want to share our contextualization of digital health message creation for a broader audience to illustrate how it is beneficial to take advantage of the various backgrounds in a multinational and multi- and interdisciplinary digital health project. Each step of the process is illustrated with a general takeaway message and lessons learned from our project.

## Methods

### Definition of Terms

In this paper, the terms health promotion, health education, health communication, health information, health literacy, health message, and digital literacy are used. Here, health education and health promotion are 2 terms that are sometimes used interchangeably [23], whereas health information and health communication are broad terms simply referring to all information and communication related to health. Moreover, health literacy can be explained as a result of successful campaigns of all the aforementioned terms, and health messages are pieces of information designed for health behavior change. In Multimedia Appendix 2 [23-32], we have provided definitions of the terms to offer the reader a more detailed explanation of their differences.

### Development of the Digital Content

#### Overview

**Figure 1:**
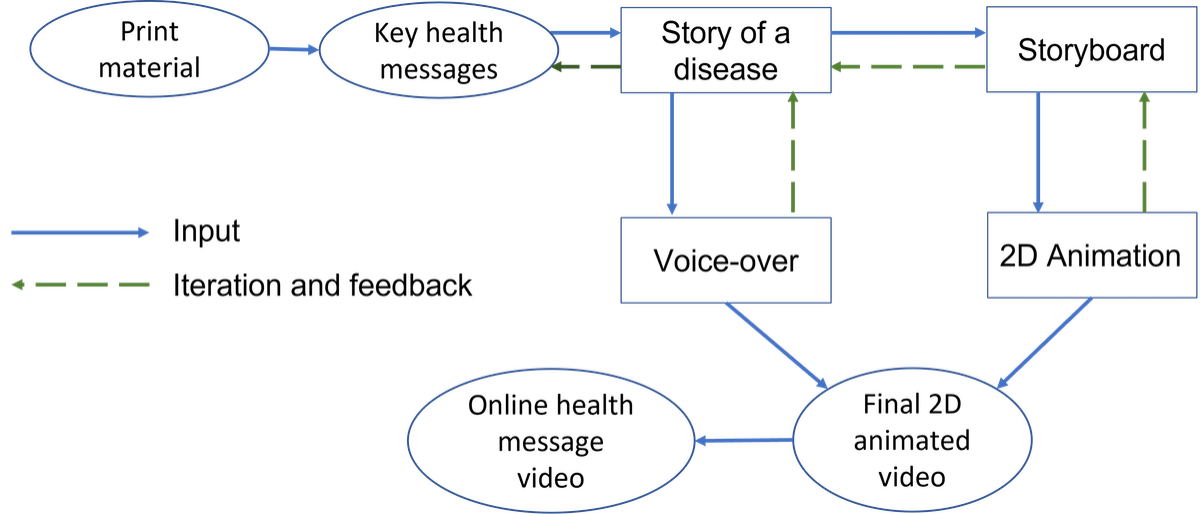
Health message digitization process.

On the basis of the information in the print materials, the digitization process began by creating a narrative story capturing the key health messages, with elements of storytelling to maintain the *clients’* attention. *Clients* is defined as “members of the public that are potential or current users of health services, including promotion activities” [33]. The stories were adapted to a rural Tanzanian village-life setting, making them valid and authentic for the clients, with an emphasis on keeping the stories simple, truthful, and emotional at times. On the basis of these imaginary stories, storyboards (drawings with captions below each image or scene) were produced to visualize the narrative so that the DigI team could provide more informed feedback. Once the storyboards were approved, the next step consisted of developing animated videos with voiceover narrations in both English and Swahili. The DigI team iterated a series of design-test-evaluate-design cycles, whereby a story was tested and improved accordingly. The digitization process is illustrated in Figure 1.

The exact approach to content creation was established based on the internal discussions. We aimed to meet clients’ needs, values, language, and culture and emphasized the rural Tanzanian context throughout the development process.

In the following 10 sections, each phase of our methodological approach is described in detail, with general advice and takeaway messages for each section.

#### A Local Needs Assessment

A human-centered design process [34] is required when developing apps to meet user requirements. In our project, a survey was conducted at the very beginning to gather users’ needs in the project villages, and a participatory and community-driven approach was deliberately preferred to the introduction of the digital health project from outside [35]. Before developing the digital health messages, several site visits were undertaken by health- and IT-researchers in the project. During these visits, local needs and wishes were gathered through discussions with local stakeholders, such as village leaders, health workers, and other community members. During the needs assessment, it became clear to the DigI team that the preferred health education format was animation. The locals explained the difficulties with low literacy in rural societies, suggesting cartoons or films for learning about health. Tanzanian Swahili was obviously a prerequisite, as the vast majority of people in Tanzania do not speak English. Although some ethnic groups have their own languages, most people understand Tanzanian Swahili. Some village stakeholders also expressed the need for other information, such as village news, village meetings, and agriculture information.

Our general advice based on our experience is to spend substantial time exploring the local needs and involving local stakeholders as partners to form the process and shape the end product from the very beginning.

#### Identification of Government-Approved Health Promotion Materials in a Nondigital Format

**Figure 2:**
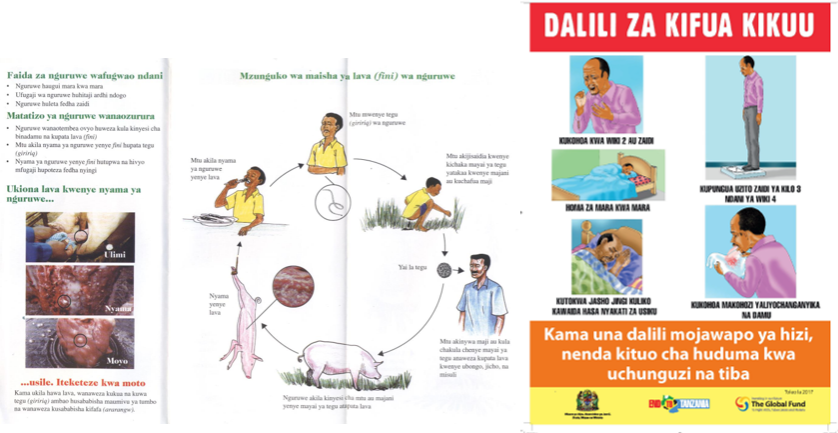
Examples of the education material used as a point of departure for digitization (Taenia solium cysticercosis and taeniasis on the left and tuberculosis on the right). Sources: Cysticercosis Working Group in Eastern and Southern Africa and Ministry of Health, Community Development, Gender, Elderly and Children, respectively.

Health strategies and guidelines may vary from one country to another, and it is important to pay attention to the efforts that have already been laid down by policy makers, health workers, and researchers at a national level when mapping the local environment. Our video animations represent up-to-date, high-quality, and clear health messages that are based on approved health information materials, as a point of departure for digitization. The messages are taken from leaflets, posters, brochures, banners, guidelines, and strategies, all carefully reviewed for key health messages aimed at a public health audience (Figure 2). We used the Ministry of Health, Community Development, Gender, Elderly and Children–approved health education messages for HIV and TB. HIV health education messages were obtained from the National AIDS Control Programme [36]. In addition, posters and presentations were obtained from the National Tuberculosis and Leprosy Control Programme, whereas the National Strategic Plan V (2015-2020) for Tuberculosis and Leprosy [37] were used as source documents. The health educational material for TSCT was provided by the Cysticercosis Working Group in Eastern and Southern Africa, and the United Republic of Tanzania One Health Strategic Plan 2015-2020 [38] was used for inspiration. The leaflets for the prevention and control of anthrax in humans and animals were jointly prepared by the Ministry of Health, Community Development, Gender, Elderly and Children, Ministry of Livestock and Fisheries, and the Ministry of Natural Resources and Tourism supported by the Food and Agriculture Organization of the United Nations and WHO country offices in Tanzania, using a One Health approach. The hard copies were converted to soft copies and shared with the DigI team for conversion into digital formats.

From this step, we recommend exercising caution and respect toward the national health authorities. National strategies and guidelines may contain important health messages to be conveyed, especially to target populations, because of context-specific reasons, and may differ from international and global guidelines.

#### Identification of Key Health Messages

Establish the most important key messages that you want to convey, depending on the health topic and target population. In our project, we dealt with infectious diseases for a rural population in Tanzania, thus dividing our key health messages into the following domains: (1) prevalence of the disease, (2) cause or transmission of the disease, (3) signs or symptoms of the disease, (4) treatment of the disease, and (5) prevention of the disease.

Once the domains were identified, we extracted key health messages from the materials described in the previous section. In the course thereof, short paragraphs, emphasizing the most important messages for each domain, were created. This is transferable to all health topics: to keep it short and simple.

The first domain intends to raise local awareness and includes health messages regarding the disease and its prevalence in the area. The second domain contains information on how diseases spread and infect others. The third domain describes the signs and symptoms of the disease, to help with early detection and management of the disease. Health messages encourage and motivate clients to seek medical advice if symptoms appear. The fourth domain is related to the treatment of diseases. The last and perhaps most important domain includes information on how people can protect themselves and their families and how the disease can be prevented from spreading between individuals and within communities.

This work established the basis for the written key health messages on the platform and in the animated health videos and moreover identified the most important information that the DigI team health task force wanted to provide to clients. The key health messages typically consisted of 50-150 words and were available on the platform. An example of a key health message (from the domain of symptoms in the TB section) is as follows:

Most TB patients show the following signs and symptoms: cough for more than 2 weeks, fever for more than 2 weeks, weight loss, night sweats and lymph node enlargement. People with these signs and symptoms should immediately visit a health facility for proper TB diagnosis and treatment.

Our general advice based on our experience is to rather focus on few key messages at the time and keep the messages short and simple so as to not overload the audience with information.

#### Creation of a Practical and Engaging Story, Easy to Understand for the General Public

Throughout time, people have learned from stories. Stories can be a powerful way to pass on knowledge and specific messages to other people [39]. In our project, we used storytelling techniques to draft a compelling everyday narrative story that clients could recognize and relate to. All animations were cooperatively developed within the DigI team; however, some partners led the process of writing the story. The draft was discussed by the DigI team. Thereafter, health content was assured by the health task force. After drafting the content of the story, our team worked on creating a word document that contained the script for each story. In our case, the script comprised facts about the disease and a creative mixture of characters, actions, and location settings within the context of a village in rural Tanzania. The scripts were carefully revised by Tanzanian DigI members to ensure local compatibility.

**Figure 3:**
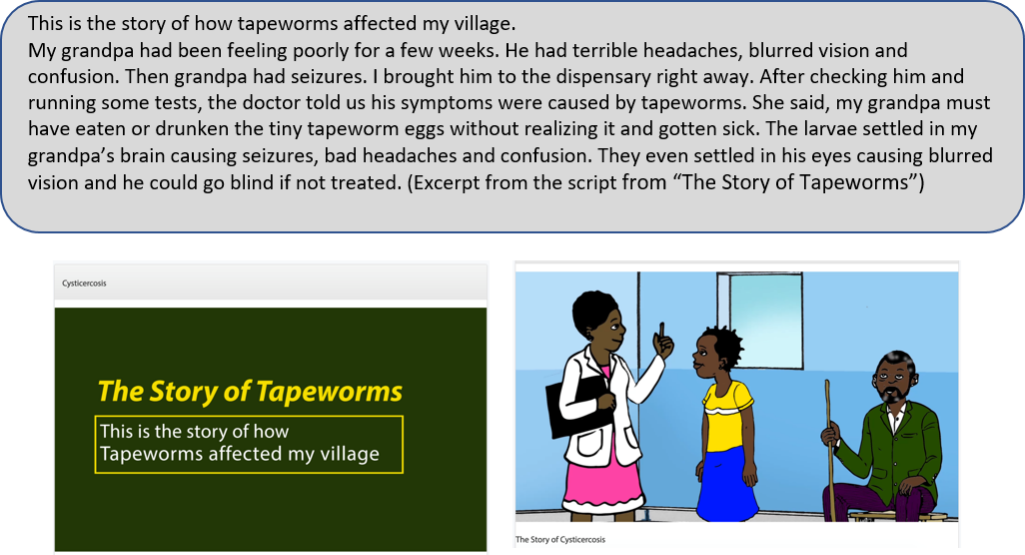
Excerpt from “The Story of Tapeworms”.

The stories are presented by a narrator and include references to family members as well as the community affected by the diseases (see TSCT example in Figure 3). The narrative includes elements to attract and maintain clients’ attention by describing everyday activities and the realities of life in a rural sub-Saharan community.

Our general advice based on our experience from the story creation is to include a variety of stakeholders to revise the scripts. Specialists and locals can contribute equally, but with different perspectives. Specialists want to ensure that the health content is being presented in an adequate way and that no key messages are left out, whereas locals can point out words and phrases that are difficult to understand. It can be useful to ask questions such as “Are all key messages promoted clearly? Is the language understandable and the story credible?”

#### Drafting of a Storyboard for an Animated Video with Review, Feedback, and Revisions

**Figure 4:**
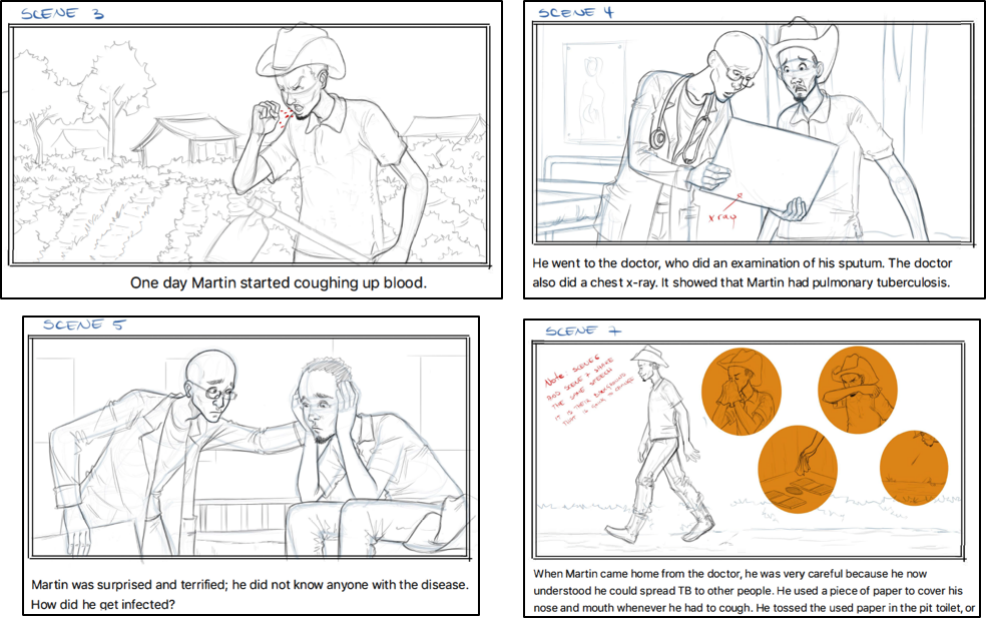
Extracts from the storyboard used in the creation of “The Story of Tuberculosis.” TB: tuberculosis.

After the script is approved, a storyboard needs to be created if the aim is to create a film or animation. A storyboard is a drawing with text below, illustrating scenes such as farming scenes that may be a common experience for rural clients (Figure 4). It is advisable to share the storyboards with the target population early on to obtain feedback in this design phase. In our project, we planned to use simplified human line drawings but were advised by the local population to incorporate more genuine characters to get the communities more involved when viewing the animations. We also received feedback from the same group to make the characters and environment more similar to those found in rural Tanzania. This included, for example, changing the façade of the health facilities and the clothes of the characters. The core health messages from the written material were left untouched, as these messages were approved, and changing them would require new ethical approval. Following an iterative process of design-test-evaluate-design, an animated video was created based on the original script, revisions, and feedback from the DigI team members and local stakeholders.

Our general advice based on our experience is to establish the storyboards as soon as the script is approved. The storyboards make the animation visualization more realistic and can be effective in identifying scenes of importance for key message uptake. It is beneficial to include target users in this stage to point out recognizability and authenticity, or lack thereof, in different scenes.

#### Forward and Backward Translation

Working in 2 languages can be challenging and may complicate the process. Hence, qualified translators with experience in the field are needed to ensure that translation is consistent throughout the various versions of the script. In our project, the health messages and story scripts were created in English and then translated to Swahili for the target audience. The process was as follows: English health messages and story scripts were given to a person to translate them into Swahili. Thereafter, the Swahili versions were given to another person to translate them back into English. Further, the back translation was compared with the original English health messages and story scripts to ensure that the messages were the same. The Swahili messages were also read by the medical doctors who spoke Swahili to ensure that the messages had the same information as the English messages.

Our experience indicated that alterations of the script were done several times, also after translation; thus, it is important to ensure that the final script represents the same key messages in both languages.

#### Audio Recording of the Story in Both Languages

When the script and translations are final, it is time to record the story. Although it is recommended to use a professional recording studio, the most important factor is to be able to present the audio file without background noise and with a clear and understandable narrator. In our project, we used professional narrators, and voiceover recordings were produced in both English and Swahili. Technically, recording itself was a straightforward process. It was emphasized that the narrators spoke slowly and clearly in an informal tone. The audio file was carefully revised by team members who were Swahili natives and near-natives in English. We recommend paying particular attention to the initial revision process of the audio file. There are cases where a written sentence conveys health messages more effectively than when reading aloud, and to save costs, it may be useful to read the script aloud to team members before recording it.

#### Finalization and Presentation of the Animation

Once the voice recordings and animation scenes are ready, the animated video can be fully produced and shared with the team for revision and improvement. In our case, all team members carefully reviewed the English version of the draft video. After at least 2 rounds of revisions, the final animated videos were produced and then presented to an audience in rural Tanzania. The *Story of Tapeworms* animation was completed in 2018. The *Story of HIV/AIDS* animation and the *Story of TB* animation were finalized in March 2019. The *Story of Anthrax* animation was completed in May 2019. The animations can be found in Multimedia Appendices 3-10.

Generally, this phase can be costly, but it is worthwhile to address small changes in the final product, which can be of importance for knowledge transfer.

#### Development of Relevant Questions Related to the Health Messages in Each Domain

**Figure 5:**
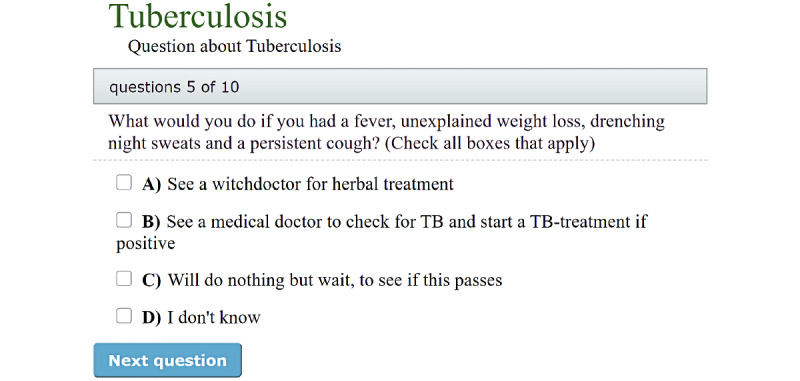
Sample questions presented on the platform. TB: tuberculosis.

To increase knowledge uptake, it may be useful to establish questions from digital health messages, such as animations, and present them to the audience in a quiz format, for example, after the audience has viewed the animation. We derived knowledge questions related to different diseases from the key health messages for each domain. These questions were constructed for research purposes to test people’s knowledge before and after being exposed to our digital health messages in a quantitative study. The same questions were published on the platform in a quiz format so that users could test their own learning. We ensured that the questions did not use medically advanced wording or information. In particular, the health task force assessed their quality as they related to the health content, and the social scientists made the questions easier to understand for the clients. An example of a quiz question is shown in Figure 5.

Our experience is that the questions created should not be too difficult, but should reflect the health messages, and work as reinforcers of the information provided in the animations.

#### Development of Web and Mobile Apps to Access the Digital Health Messages

Access to information can be a bottleneck in disease prevention in the Global South. Animations and other digital formats for health education in the local language are only useful if they are accessible to the target audience. Hence, in the DigI project, it was equally important to develop an easy-to-use digital platform so that people in rural areas could access health messages. Key requirements for the success of the platform included that it would be easy to learn *how to use* it and that it could be used with a basic level of digital literacy (Multimedia Appendix 2). Given that most people are likely to access health messages on a mobile phone, we adopted the *mobile-first* design approach, creating a responsive web application and an Android-based mobile app. The web application presents health messages interactively, whereby a user is presented with an animated video, key health messages, graphics, and a quiz.

As part of the human-centered design process [34], the project team specified the requirements of web and mobile app systems based on inputs from village stakeholders in Migoli and Izazi in the Iringa region. The proposed designs were iteratively improved from a conceptual design to low-fidelity prototypes and high-fidelity interactive prototypes using Adobe XD [40]. The low-fidelity prototypes were simple sketches and wireframes to present early user interface designs for discussions in the DigI team. These helped in gathering user feedback and suggestions on what could be improved. Furthermore, high-fidelity prototypes presented more enriched user interfaces, with interactivity for the test users to experience the interaction with the designed tool. In addition to testing and evaluation by the medical task force and other team members in Tanzania, user-based testing was conducted using the IT task force to uncover potential usability problems. User testing focuses on efficiency, effectiveness, and overall user satisfaction. Using purposive sampling, a total of 12 test users, aged 15-25 years, participated in 2 rounds of usability testing conducted in Kigali (Rwanda) as part of an iterative design process. All test participants had postprimary school education, but none had completed secondary school. The first high-fidelity prototypes were tested by 8 participants (3 women and 5 men) in October 2018, and the second improved versions of the web and mobile app were tested by 4 participants in July 2019. The findings indicated that it was easy to access health information on the platform, view the videos, and read key health messages for different diseases. Test users reported that it was easy to navigate the system and that the content was logically structured. However, some respondents said that “it was hard to find the videos” in the early versions of the platform. Hence, global navigation was redesigned, adding on the top menu a direct link to a page with videos for all diseases. Test participants also suggested making two separate videos for each disease: one video for a story and another with only the health messages. However, after deliberations, the project team decided to keep 1 integrated animation per disease, not too long to keep the audience’s attention. Furthermore, test users suggested additional requirements, such as the possibility of self-diagnosis through search functionality on symptoms. This indicated the user’s perception of the platform as a health system. Subsequently, we used design constraints to prevent users from attempting to do anything that the system is not intended for, to make clear that the goal of the platform is to provide health information rather than medical diagnosis. It is in this regard that global navigation includes links to *Health Information*, including videos, text, and quizzes, in addition to a contact form, but there is no search bar. However, given the small screen size of mobile devices, the mobile app includes a search field to help users easily access information on the diseases included in the platform.

**Figure 6:**
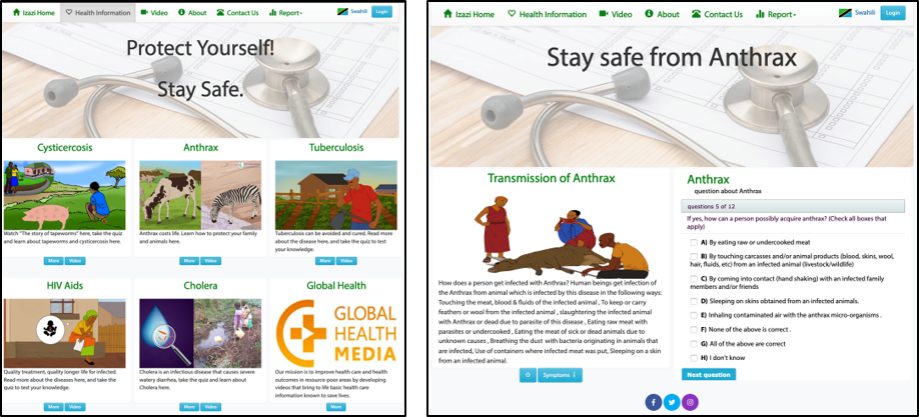
The platform user interface. The dashboard on the main health page is shown on the left. On the right, an example of the key health messages and quiz questions within the transmission domain of Anthrax is portrayed.

The test participants were given a number of tasks to carry out, while a usability evaluator observed their performance to find any problems with the software under testing. Although posttest focus group interviews were conducted to gain insights into users’ impressions, a self-reporting web-based survey helped to collect users’ opinions on a Likert scale. The resulting web designs, shown in Figure 6, have been deployed on village servers so that clients can access health messages [22] from an InfoSpot free of charge, or on the internet. The viewer can explore video animations, key health messages, and quizzes when clicking on the different diseases. The cholera animation was developed by The Global Health Media Project before the DigI project and was included on the platform as an add-on. The dashboard also provides the user with an option to view more videos.

**Figure 7:**
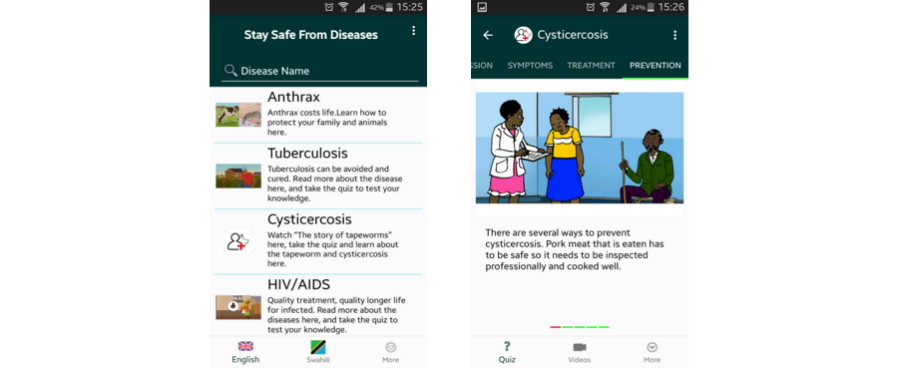
Health Messages mobile app "Linda Afya".

Given that an increasing number of people have access to low-cost mobile devices, an Android mobile app, shown in Figure 7, was also created to present the same health messages as on the platform. Mobile app users have the option to download all the videos on their devices so that they can view them without an internet connection or being close to the InfoSpot. One of the possible scenarios for using the app is that someone visits an InfoSpot with Wi-Fi, then installs the app and downloads all videos for later viewing together with the family back home.

On the basis of our experience, we recommend digital health content developers to assess the access and context that users have to any digital information source. Digital health education projects are only useful if the target population is able to access health messages either supervised or unsupervised.

## Results

**Figure 8:**
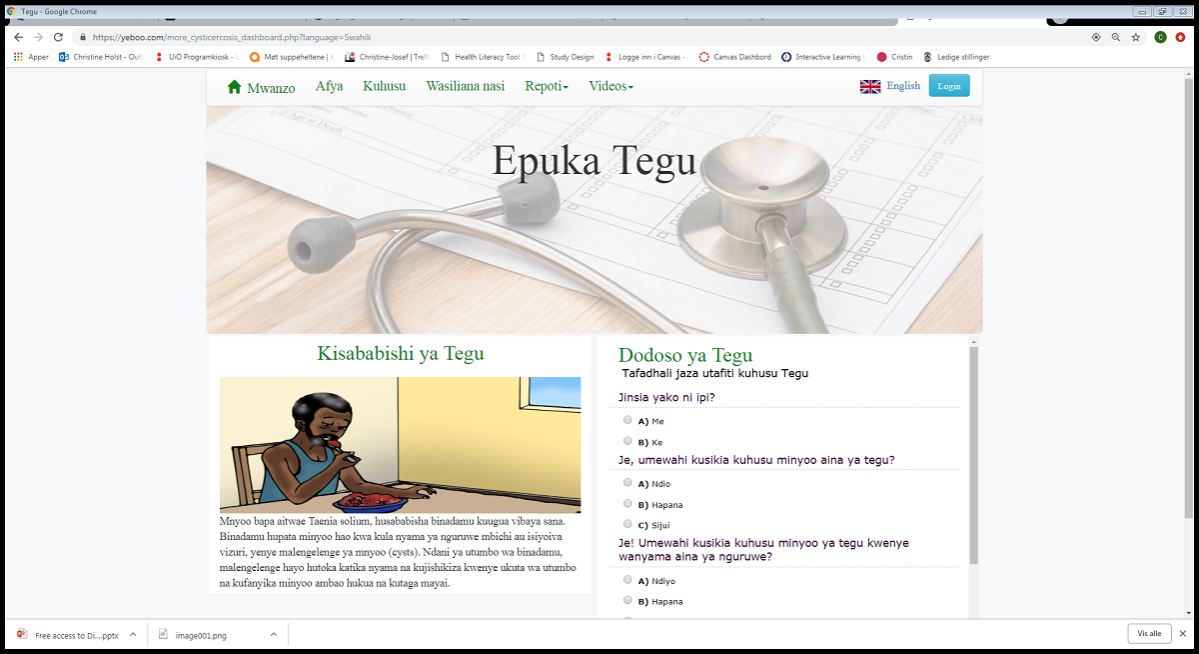
Swahili Taenia solium cysticercosis and taeniasis health education on the platform.

The resulting apps developed through the above-described process can be found via the internet [22] or accessed via Wi-Fi in InfoSpots using locally stored village servers in rural Tanzania. When accessing the platform, the client is introduced to diseases of public health importance in Tanzania (as shown in Figure 6). At any time, the client can change the language from English to Swahili or from Swahili to English. The diseases are HIV/AIDS, TB, TSCT, and anthrax, with all digital information developed as part of the DigI project. In addition, users can find more videos on a variety of health topics, including a cholera animation. The client has 2 choices per disease: *Video* and *More*. When clicking on the *Video* button, an animated video starts immediately. When clicking on the *More* button, the client is taken to a page addressing the specified disease. The client can then navigate through the 5 domains, read about the key health messages, and take the quiz for each disease. Graphics from the animations are used as illustrations for the domains (Figure 8).

In November 2019, seven InfoSpots were available and accessible with the platform, in Esilalei, Selela, Migoli, and Izazi. InfoSpots are still accessible and available for the local population. As of September 3, 2020, the mobile app had been installed by 425 users in 3 months since the launch of the latest version in June 2020. By February 1, 2021, the app store listing had been visited by 1531 people, out of which 1127 were users registered in Kenya. The app can be installed worldwide by searching the app called *Linda Afya* on the Google Play app store.

## Discussion

### Principal Findings

In this paper, we have provided a tutorial for the various steps in the development process of digital health messages for a rural population in sub-Saharan Africa. The result of our project is a functional health education platform, and the feedback from the local stakeholders and test users was included to improve the platform’s usability and impact for the target users. In this section, we present a discussion of the chosen approach used in this project.

A multi- and interdisciplinary approach has become increasingly important in complex research and innovation projects [41]. The advantage of multi- and interdisciplinary collaborations is the ability to draw from each other’s knowledge, perspectives, and experiences and channel it toward a joint objective. In this project, the collaboration among team members with medical, social sciences, and IT backgrounds was especially beneficial. Throughout, close collaboration among the local and international health experts, translators, and village stakeholders was important for good results. Furthermore, the storyboard, animated videos, web-based platform, and mobile app could not have been created without input from the visual designers and IT task force. The high quality of digital health messages could not have been achieved without advice from the Tanzanian members of the health task force. International advice on local health matters may differ from the national health advice. Hence, local health researchers were also irreplaceable in identifying the guidelines and written government-approved materials related to each disease. When identifying the key information and creating short, clear messages for the basic health promotion material, the team benefited from members with experience in communication and linguistics. This was important because medical doctors, researchers, and health workers tend to use language with terminology that is difficult for the general public and patients to understand, which can have an exclusive effect [42].

A mixed methods study is currently ongoing within the DigI project to gather evidence for health information uptake and retention based on the above-described animations and access to the platform in 2 villages in rural Tanzania [43]. The data are currently being analyzed, and results are expected late this year. This evaluation will play an important role when revising key messages to become even clearer for the local population. By performing iterations in response to feedback from local communities over time, a human-centered design of global health education is emphasized with its ultimate goal of empowering communities [12].

Regarding the technical aspects of the animation development process, there are various lessons learned that we want to share. We started with the identification of relevant key health messages and then created local stories around those messages, as described above. We had wishes and recommendations from the local community that increased the technical difficulty level but were crucial for the reception in the communities. The many inputs from local stakeholders were acted upon as the DigI team emphasized a co-design process.

Feedback from people in rural areas indicated that the proposed platform should provide information in three main categories: (1) village information, (2) health information, and (3) information on social life and activities. On the basis of this, the design team created templates and user interfaces. In the next stage of the co-design process, people were presented with the interfaces, and they gave positive feedback that the platform was easy to use. However, it seemed difficult to collect the necessary information for publication on the platform, particularly concerning the villages’ information and social information. The involved users had limited digital literacy for creating and updating information, for example, using the social media feeds provided by the platform. The research team realized the need to work with a selected group of individuals in each village to increase the usefulness and acceptance of the platform.

The inclusion of digital literacy training programs for the local population is a key factor in the successful implementation of digital health projects. Undoubtedly, the importance of digital literacy is evident when it comes to the use of digital health technologies by people with lower literacy levels, as these technologies could help overcome limitations and surely include new groups in the information society. The DigI team also set up the Key Performance Indicators framework for digital society development and, specifically, to provide a success indicator framework for this project. One of the most relevant success indicators is the level of digital literacy skills, and the health knowledge retention of the participants after the digital skills programs were obtained. Digital literacy goes hand in hand with digital inclusion and social empowerment; thus, it is important that Key Performance Indicators become an integral part of digital literacy initiatives and projects [24].

The drafted stories seemed only to meet the agreement of the full team after several rounds; therefore, the iterative process went on for a long time. In retrospect, the drafting of the storyboards should have been done at an earlier stage within the project, as this process would have provided the group with a good overview of the key health messages and scenes that we wanted to disseminate to the local population. It would have been much easier to imagine the final result if it had been possible to review the different scenes of the animation at an earlier stage.

The translation process of the first animation (TSCT) was performed too early. As the DigI team worked in English and the editing process went on over time, we had to update the Swahili version several times. When the final script was completed in English, the translators had to go through the Swahili version again to ensure that the 2 scripts convey the same story. Consequently, at the stage of the actual recording, the TSCT animation had to be recorded twice because the spoken version sounded different from how it was read. We also had to make adjustments to some wording to avoid misunderstandings. Rerecording was costly and time consuming and could have been avoided. In the following productions, we were more careful with the voiceover recording in the working language and the finalization of the script before translation.

The IT, social science, and health researchers in the DigI project undertook a participatory research approach and spent substantial time partnering with the local population to identify the preferences for digital health message design and knowledge gaps. The WHO has recommended a similar approach of using the existing Ministry of Health documentation and the engagement of different stakeholders [44]. Members of the community, including health workers, school teachers, village leaders, and community leaders, are also key stakeholders to be involved through discussions and interviews to prepare appropriate and relevant health messages and other information. It is essential to engage communities through representatives, including community leaders, to cocreate digital content but also to reach expert consensus on health messages after receiving feedback from the communities. Another important aspect to be considered is user adoption of the messages, whereby health messages are expected to be disseminated to, and accessed by, all or a large proportion of the targeted audience via the platform.

A study that introduced a remote measurement technology platform reported that during the introduction of the remote measurement technology, a multi-stakeholder approach, including patients and research clinicians, provided knowledge about varying requirements for the design and development of the platform [45]. The involvement of patients empowered them to understand the value of the system and to provide their views and needs, thus facilitating the development of a user-adapted platform. Furthermore, the need has been emphasized for developers of digital health information to use numerous innovative strategies to meet both the needs and expectations of targeted audiences [46]. During consultative meetings in the early phases of our project, village leaders indicated the need to use the platform to disseminate news and information to villagers. This included schedules for village meetings, agricultural information on the market, and prices of their products, which were initially not foreseen by the experts. This once again indicates that it is paramount to involve the right people at the right time in an iterative manner to co-design information to be included in an intervention [47]. This multi- and interdisciplinary approach allows for continuous improvement to meet the needs of the targeted audiences.

### Conclusions and Future Plans

The digital health messages, video animations, quizzes, and web applications described in this paper were created through an integrated approach based on various scientific disciplines in addition to engagement of and input from village stakeholders and test users, thus taking advantage of the network and expertise of the DigI team members.

We believe that the methodological approach described in this paper, referring to the digitization of approved printed health education materials, should be promoted. The production process resulted in high-quality educational material that can be used in different forms and in different settings, such as stationary health messages on an internet platform or animated videos telling the story of four diseases in their local context in rural Tanzania. Potentially, health animations developed through the approach described in this paper could also be used in national knowledge portals, as the health messages they contain have been fully approved.

## Appendix

Multimedia Appendix 1 Screenshot of the platform.

Multimedia Appendix 2 Definition of terms.

Multimedia Appendix 3 HIV/AIDS health animated video (English).

Multimedia Appendix 4 Tuberculosis health animated video (English).

Multimedia Appendix 5 Taenia solium cysticercosis and taeniasis health animated video (English).

Multimedia Appendix 6 Anthrax health animated video (English).

Multimedia Appendix 7 HIV/AIDS health animated video (Swahili).

Multimedia Appendix 8 Tuberculosis health animated video (Swahili).

Multimedia Appendix 9 Taenia solium cysticercosis and taeniasis health animated video (Swahili).

Multimedia Appendix 10 Anthrax health animated video (Swahili).

## Abbreviations

DigI: Non-discriminating access for Digital Inclusion
InfoSpot: information spot
IT: information technology
TB: tuberculosis
TSCT: Taenia solium cysticercosis and taeniasis
WHO: World Health Organization
