# Supplementary figures and images for: Development of Digital Health Messages for Rural Populations in Tanzania: Multi- and Interdisciplinary Approach
Source: JMIR Mhealth Uhealth. 2021 Sep 22;9(9):e25558. doi: 10.2196/25558 (PMC8495580; doi:10.2196/25558)

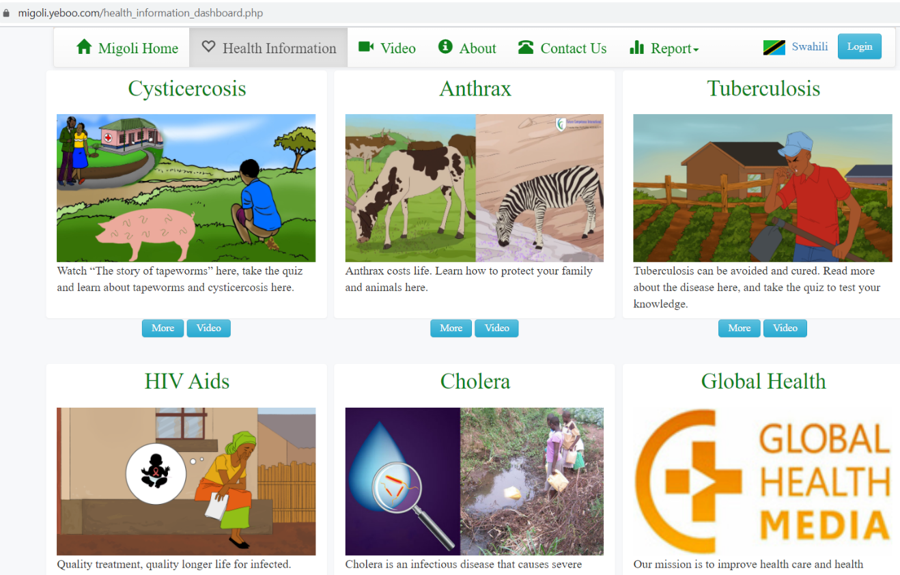

Supplement: Multimedia Appendix 1 [file mhealth_v9i9e25558_app1.png]
