# Supplementary material for: Development of Digital Health Messages for Rural Populations in Tanzania: Multi- and Interdisciplinary Approach
Source: JMIR Mhealth Uhealth. 2021 Sep 22;9(9):e25558. doi: 10.2196/25558 (PMC8495580; doi:10.2196/25558)
Supplement: Multimedia Appendix 2 [file mhealth_v9i9e25558_app2.docx]

# Multimedia Appendix 2. Definition of terms

### Health promotion

Health promotion has a wide meaning, and was defined by the Ottawa charter in 1986 as “*the process of enabling people to increase control over, and to improve, their health”* [1]. The term relates to social and political processes, aiming to strengthen skills and capabilities of individuals to impact public and individual health [2]. With today’s channels for self-learning through digital tools, health promotion has changed its face and has become increasingly important in disease prevention (primary, secondary & tertiary), management and early treatment.

### Health education

Health education is seen as an essential component of action to prevent diseases and promote good health [3]. It relates to providing health information to an audience, aiming to increase health knowledge, so that people voluntarily adopt healthy behaviours [4]. In the World Health Organization’s “Health Promotion Glossary” the term is defined as follows: “*Health education comprises consciously constructed opportunities for learning involving some form of communication designed to improve health literacy, including improving knowledge, and developing life skills which are conducive to individual and community health.”*  [2]

### Health communication

Health communication includes various areas, for example edutainment, health journalism, interpersonal communication, media advocacy, organizational communication, risk communication, social communication and social marketing [2]. The term was described by the PAHO (1996) as a key strategy to inform the public about health concerns and to maintain important health topics on the public agenda, and point out the use of mass and multimedia and other technological innovations to share health information to increase awareness of specific aspects of both individual and collective health [5].

### Health information

Health information is probably the term in this context that is the least defined. It is often related to clinical aspects: “*Health information is the data related to a person’s medical history, including symptoms, diagnoses, procedures, and outcomes.”* [6] However, health information covers also information about population health or other health-related issues such as health education, promotion and statistics required for planning and decision making. Health information can thus be any information about personal or population health.

### Health literacy

Health literacy is a term used to describe people’s abilities to engage with health information and services [7]. The term was applied in the 1970’s, and received increased attention related to health promotion practice during the 1980’s. When people are adopting health knowledge, i.e. after being exposed to health promotion or health education campaigns, their health literacy increases. WHO defines health literacy as a construct that “represents the cognitive and social skills which determine the motivation and ability of individuals to gain access to, understand and use information in ways which promote and maintain good health.” [2]

### Health message

A health message is a persuasive message, designed for health behaviour change [8]. The health messages can be put across to people in two ways; directly by person-to-person, or indirectly by a mediator like radio, TV [9], or digital formats.

### Digital literacy

Digital literacy is the set of skills and the ability to access, manage, understand, integrate, communicate, evaluate and create information and multimedia safely and appropriately through digital devices and networked technologies for participation in educational, economic, and social life. It includes 21^st^ century competencies that are variously referred to as computer literacy, ICT literacy, information literacy, participation literacy, online literacy, and media literacy. Digital literacy, as seen in most high-income countries, incorporates multiliteracies and multimodal dimensions—which are the technical, cognitive, and social-emotional dimensions [10].

## References

1. The Ottawa Charter for Health Promotion. World Health Organization. 1986. URL: https://www.who.int/teams/health-promotion/enhanced-wellbeing/first-global-conference [accessed 2021-08-26]
2. Nutbeam D. Health promotion glossary. Health Prom Int 1998 Jan 01;13(4):349-364. [doi: 10.1093/heapro/13.4.349]
3. Nutbeam D. Health literacy as a public goal: a challenge for contemporary health education and communication strategies into the 21st century. Health Prom Int 2000;15(3):259-267. [doi: 10.1093/heapro/15.3.259]
4. Kumar S, Preetha G. Health promotion: an effective tool for global health. Indian J Community Med 2012 Jan;37(1):5-12. [FREE Full text] [doi: 10.4103/0970-0218.94009] [Medline: 22529532]
5. Cerqueira MT, Coe GA. Communication, Education and Participation: a Framework and Guide to Action. Washington, D.C: Pan American Health Organization; 1996.
6. What is Health Information? The American Health Information Management Association (AHIMA). URL: https://www.ahima.org/certification-careers/certifications-overview/career-tools/career-pages/health-information-101/ [accessed 2021-08-26]
7. Dodson S, Good S, Osborne RH. Health literacy toolkit for low- and middle-income countries: a series of information sheets to empower communities and strengthen health systems. New Delhi: World Health Organization, Regional Office for South-East Asia, 2015. URL: <https://apps.who.int/iris/bitstream/handle/10665/205244/B5148.pdf?sequence=1&isAllowed=y> [accessed 2021-08-26]
8. Morrison FP, Kukafka R, Johnson SB. Analyzing the structure and content of public health messages. AMIA Annu Symp Proc 2005:540-544 [FREE Full text] [Medline: 16779098]
9. World Health Organization. Education for Health: A Manual on Health Education in Primary Health Care. Geneva: World Health Organization; 1988:1-261.
10. Radovanović D, Holst C, Belur S, Srivastava R, Houngbonon G, Le Quentrec E, et al. Digital literacy key performance indicators for sustainable development. Soc Inclusion 2020 May 14;8(2):151-167 [FREE Full text] [doi:10.17645/si.v8i2.2587]
